# Cancer-related fatigue and activities of daily living: lessons learned from the COVID-19 pandemic

**DOI:** 10.1186/s12904-024-01437-z

**Published:** 2024-04-27

**Authors:** Iveth Urbano Chamorro, Julio C. de la Torre-Montero

**Affiliations:** 1 Universidad Pontificia Comillas, Health Sciences Department, Madrid, Spain; 2 Facultad de Ciencias de la Salud, Universidad Rey Juan Carlos, Madrid, Spain; 3 Fundación San Juan de Dios, Madrid, Spain

**Keywords:** SARS-CoV-2, COVID-19, Pandemic, cancer-related fatigue, Fatigue, Symptoms, Activities of daily living, Quality of life

## Abstract

**Background:**

Cancer-related fatigue is a prevalent condition in all stages of oncologic disease that is poorly diagnosed, with a negative impact on physical function to perform activities of daily living. Fatigue is also one of the main manifestations in post-COVID-19 syndrome, and few studies have explored the functionality of cancer patients after infection by the new coronavirus. This study was designed to assess cancer-related fatigue symptoms and their implications on physical function and quality of life during the pandemic.

**Methodology:**

An observational study with a cross-sectional survey in cancer patients ≥ 18 years of age was conducted. The Functional Assessment of Chronic Illness Therapy - Fatigue Scale (FACIT-F), the perception of asthenia and performance status were evaluated, and the differences between groups according to the history of COVID-19 were calculated.

**Results:**

A total of 60 cancer patients had an average age of 33.5 ± 10.11 years, 73.3% were female, and 98.3% had an Eastern Cooperative Oncology Group-Performance Status level < 2. Severe fatigue was found in 43.3% of patients, and the average FACIT-F score was 33.5 ± 10.11. The proportion of coronavirus infection was 13,3%, and the performance of this group was worse on the scale compared to the group without infection (25 ± 10,40 vs. 34,81 ± 9,50 [*p* = 0,009]). There was a significant correlation between visual analog scale values and FACIT-F scale scores (Pearson’s *r* = -0.76).

**Conclusion:**

SARS-CoV-2 infection could increase cancer-related fatigue symptoms, limiting activities of daily living and impairing quality of life.

**Supplementary Information:**

The online version contains supplementary material available at 10.1186/s12904-024-01437-z.

## Introduction

The most common symptom experienced by oncology patients is fatigue, referred to as Cancer-related fatigue (CRF), it is currently defined as a distressing, persistent, subjective sense of physical, emotional, and/or cognitive tiredness or exhaustion related to cancer or cancer treatment that is not proportional to recent activity, does not improve with rest and interferes with usual functioning [1, 2]. The estimates of CRF prevalence an overall pooled is 52% [3], variable throughout all the cancer trajectory from diagnosis to the end of life [4], and one-third of survivors report CRF as persistent fatigue for several years after treatment [5]. In Spain, 75% of unsuccessful return to work in cancer patients is related to CRF [6]. Also, patients undergoing active therapy are more likely to report more severe symptoms and an incidence of CRF up to 90%, since it can be considered one of the main side effects of some antitumor therapies [7].

Despite the epidemiology, there is not a complete understanding of CRF pathophysiology, beside the relation with treatments, psychosocial, behavioral, and biological factors. In the last group, a variety of tumor mechanisms have been investigated, including proinflammatory cytokine release, neuroendocrine dysregulation, prolonged alterations in the cellular immune system and disruption in muscle energy metabolism [8, 9]. Other potential contributing factors are involved, such as anemia, depression, sleep disorders, malnutrition, cardiopulmonary diseases, and hypothyroidism [10, 11]. Therefore, CRF is a common cause of impaired physical function and autonomy to perform activities of daily living (ADL) [4, 12]. Additionally, preserved physical function is a good predictor of the life expectancy of cancer patients [13, 14], and evidence indicates that the functional capacity for ADLs is of extreme importance for the preservation of health-related quality of life (HRQoL) and should be optimized during treatment and palliative care interventions [15, 16].

In order to this, there is accordance among guidelines that all cancer patients should be screened for the presence of fatigue symptoms and establish an opportune intervention. As a subjective experience, CRF is measured most efficiently via self-report; unidimensional scales, such as VAS (visual analog scale) or the Brief Fatigue Inventory (BFI), are the best screening tools in the clinical context [2]. The multidimensional scales are complex but cover more fatigue aspects and meet accepted standards of validity. The most widely used are the European Organization for Research and Treatment of Cancer quality of life questionnaire fatigue subscale (EORTC QLQ C30), Multidimensional Fatigue Inventory (MFI-20), and Functional Assessment of Chronic Illness Therapy - Fatigue Scale (FACT-F) [17].

Nowadays, the pandemic caused by severe acute respiratory syndrome coronavirus 2 (SARS-CoV-2) focuses health services on vulnerable populations, such as cancer patients, who have an increased risk of infection and develop severe complications or even death [18], especially those with advanced tumor stage or low performance status [19]. Cancer patients also suffer indirect complications in response to the detrimental impact of COVID-19 on cancer care centers worldwide, where some centers estimated that up to 80% of their patients were exposed to harm due to reduced services as part of a preemptive strategy (55.34%), overwhelmed system (19.94%), staff shortage (17.98%), restricted access to medications (9.83%) [20], and reduced outpatient visits and social issues [21].

In Spain, there is no official epidemiological report on COVID-19 in the oncology population; nevertheless, there are studies that offer data on the impact of COVID-19 on Spanish cancer patients during the pandemic, for instance, there has been a 20.8% decrease in newly diagnosed cases [22]. Previous investigations into the post-COVID-19 period have predominantly focused on the general population, with a notable emphasis on the prevalent and frequently reported symptom of fatigue [23]. Furthermore, post-COVID-19 manifestations are expected to have a negative impact on long-term quality of life, especially in young adults [24]. Due to this, post-COVID syndrome is now defined, also known as long COVID, characterized by residual signs and symptoms that persist or develop 4 to 12 weeks after the onset of the acute illness. These symptoms cannot be explained by an alternative diagnosis, and as of now, the pathophysiology remains unclear [25].

This work was initiated owing of the lack of data regarding the impact of COVID-19 on CRF, also, studies inolving oncological populations are needed as a health strategy to address this global situation promptly. For this reason, the main objective of this study was to assess CRF symptoms and their implications on functional capacity for ADLs and HRQoL in cancer patients during the COVID-19 pandemic, as well as to evaluate whether patients who had a previous infection perceived more impairment in HRQoL.

## Materials and methods

### Design

A retrospective observational study was conducted between February and May 2021 in cancer-diagnosed patients in Spain, with a cross-sectional measurement of CRF, physical function on ADLs and HRQoL through an online questionnaire. Before data collection, a pilot with a provisional version of the questionnaire was made in 4 participants (an oncologist, a cancer patient and two people without oncological disease) to identify the response time and make some editorial changes based on their feedback, these preliminary results were not used for the final analysis.

### Participants and settings

A sample size was calculated based on a cancer prevalence of 2% according to the Spanish Society of Medical Oncology (SEOM) 2020 report [26], with a confidence level of 99%, an alpha error of 5% and 10% of planned replacements. The study population was recruited through social media and cancer foundations/associations; then received an electronic link that directed them to a brief explanation of the study and the survey prior to consent.

**Fig. 1:**
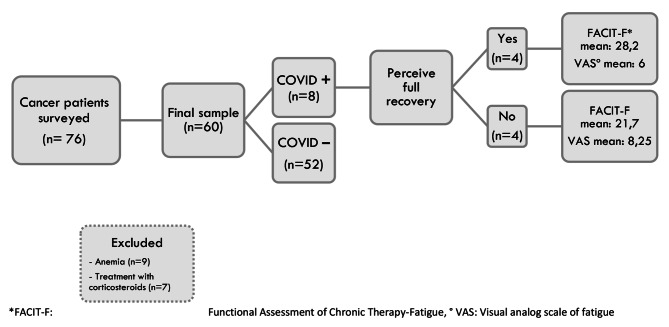
Patient flow chart. *FACIT-F: Functional Assessment of Chronic Therapy-Fatigue, ° VAS: Visual analog scale of fatigue

Participants ≥ 18 years of age with active cancer diagnosis of any etiology were included and completed the survey. Individuals with altered cognitive status or neurological diseases that impaired the question response, anemia, ECOG levels 4 (confined to bed completely disabled, totally confined to bed) and 5 (dead), and active use of available pharmaceutical options for CRF [27], were excluded (Fig. 1). The application of the online questionnaire and the verification of the information were carried out over a period of three months (February 22nd and May 23rd of 2021), and the data of the final sample were subsequently encoded in a database for analysis.

### Measuring instrument

A questionnaire was designed for this study and was organized in two sections:

In the first section, sociodemographic and clinical characteristics were collected, including whether there was a history of COVID-19 detected by any method, its characteristics and the patient’s perception recovery after infection. The second section contains CRF variables:

- The symptom level of CRF was measured by VAS (visual analog scale) on a numerical rating scale from 0 to 10, where a score between 1 and 3 corresponds to mild fatigue, 4 to 6 to moderate fatigue and 7 to 10 to severe fatigue, and a score = 0 corresponds to the absence of the symptom.
- The FACIT-F (Functional Assessment of Chronic Illness Therapy - Fatigue Scale) is a self-administered scale to assess perceived fatigue and its impact upon physical function during usual daily activities over the last 7 days and is commonly used in cancer populations. This instrument is a 13-item questionnaire, each item was rated on a 0–4 Likert scale, the total score can range from 0 to 52, where a higher score indicates a better HRQoL despite CRF. A score of ≤ 30 indicates severe CRF symptoms. The FACIT-F scale has been validated in many studies and is available in Spanish [28]. For the purposes of this study, a license was acquired.
- To determine the functional ability to perform ADLs, we used the Eastern Cooperative Oncology Group-Performance Status (ECOG-PS). This scale was developed in 1960 and is a discrete quantitative variable that describes patients’ level of functioning in terms of their ability to care for themselves, daily activity, and physical ability, whose values are in a range of 0 (fully activated state) to 5 (dead). Its validity and reliability have been investigated in several studies and widely used in clinical practice [13]. In the questionnaire, we used levels 4 and 5 as exclusion criteria. In the questionnaire, we used levels 4 and 5 as exclusion criteria due to the functional deterioration involved and the inability to respond to the questionnaire (Level 4: Completely disabled. Cannot carry on any self-care. Totally confined to bed or chair, and Level 5: Dead).

This digital survey was applied by Microsoft 360 Forms®, available for any device with internet access.

### Data analysis

The analysis of the database was performed using IBM Corp. Statistical Package for the Social Sciences Released 2020 (SPSS) for Windows, Version 27.0. Armonk. Initially, an exploratory evaluation of the data was carried out, and a total of 6 missing values ​​were discarded.

A descriptive analysis was performed in which measures of central tendency and dispersion were used for quantitative variables and categorical variables were described as percentages, graphs, and frequency tables. The results from FACIT-F as a dependent variable were compared in different groups, one of these according to the history of SARS CoV-2 infection, this magnitude of association was evaluated based on the type of variable under comparison. For quantitative variables in two independent groups a t -test was used, and for categorical variables of more than one group an ANOVA test was performed. A p value of < 0.05 was considered to indicate statistical significance. The correlation coefficient and its significance between the FACIT-F and VAS fatigue results were determined using Pearson’s test and represented by a scatter plot.

The STROBE Statement checklist for cross-sectional studies was used for this report [29].

## Results

### Sample characteristics

A total of 76 oncological patients answered the online questionnaire between February 22nd and May 23rd of 2021, and the average completion time was eight minutes. After excluding 16 participants, a final sample of 60 patients was analyzed in our study, among these 13.3% (*n* = 8) had coronavirus infection (Fig. 1).

Sociodemographic characteristics of the participants are summarized in Table 1. Most patients were female (73.3%), and the median age was 54.7 years old [IQR 33–81]. The main place of medical care center was the urban area; 88.3% of patients lived with their families, 11.7% lived alone, and no patient was living in a nursing home. Only 28.3% were actively working during the study. Most of the patients (88.35%) perform some level of physical activity, most of these for at least 150 min per week.

**Fig. 2:**
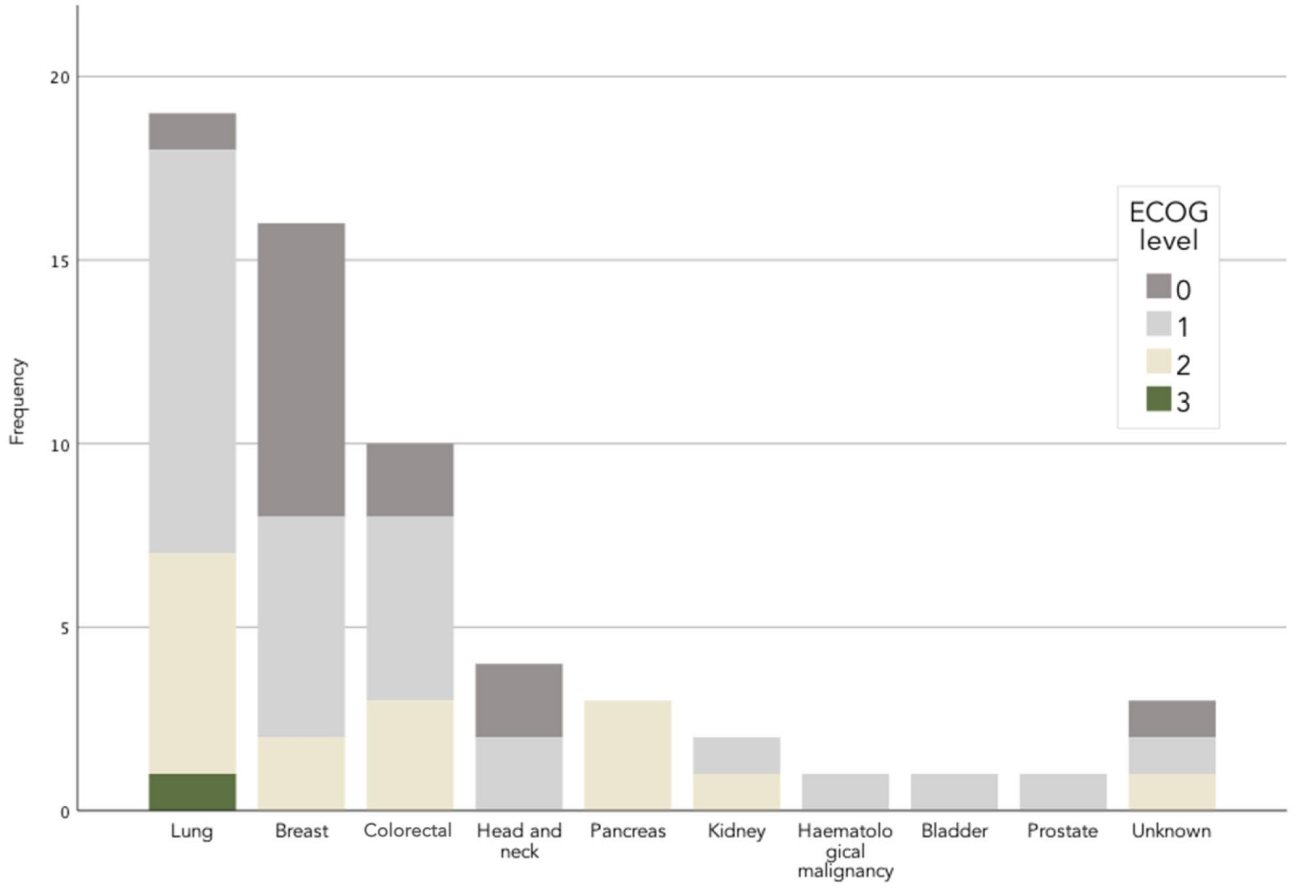
ECOG performance status by cancer type

In our sample, the most common types of primary cancer were lung (31.7%), breast (26.7%) and colorectal (16.7%). A 43.3% of the total had metastatic disease, especially the group with lung carcinoma (18.3%) (Fig. 2; Table 2).

**Table 1:** Demographic and clinical characteristics

All (*N* = 60)	
**Age (years)**	
Mean ± SD	54,7 ± 10,52
Min - Max	33–81
Sex, n (%)	
Female	44 (73,3)
Male	16 (26,7)
**Place of medical care, n (%)**	
Urban	52 (86,7)
Rural	8 (13,3)
**Living arrangements, n (%)**	
Lives with family	53 (88,3)
Lives alone	7 (11,7)
**Employment status, n (%)**	
Retired	21 (35)
Currently working	17 (28,3)
Studying	14 (23,3)
Not employed	8 (13,3)
**Physical activity, n (%)**	
≥ 150 min/week	32 (53,35)
< 150 min/week	21 (35,0)
None	7 (11,7)
**Comorbidities, n (%)**	
Hypothyroidism	8 (13,3)
Chronic pulmonary disease	3 (5,0)
Depression	2 (3,3)
**ECOG performance status, n (%)**	
0	14 (23,3)
1	29 (48,3)
2	16 (26,7)
3	1 (1,7)

SD: Standard deviation

**Table 2:** CRF results by Cancer type

Primary tumor location		Metastatic disease, *n* (%)		FACIT-F			VAS of CRF
*n* (%)		Yes	No	Mean ± SD	CI (95%)	Range	Mean (range)
Lung	19 (31,7)	11 (18,3)	8 (13,3)	33,37 ± 9,71	28,69–38,05	17–52	5,32 (0–9)
Breast	16 (26,7)	2 (3,3)	14 (23,3)	32,88 ± 10,99	27,02–38,73	11–49	5,88 (1–8)
Colorectal	10 (16,7)	4 (6,7)	6 (10)	37,60 ± 9,26	30,97–44,23	20–51	4,20 (0–9)
Head and neck	4 (6,7)	1 (1,7)	3 (5)	38,50 ± 5,91	29,09–47,91	33–45	5,00 (1–7)
Pancreas	3 (5,0)	3 (5)	0	18,00 ± 3,60	9,04–26,96	14–21	7,67 (6–9)
Kidney	2 (3,3)	2 (3,3)	0	27,50 ± 7,77	-42,38–97,38	22–33	7,00 (6–8)
Hematological	1 (1,7)	0	1 (1,7)	35,00 --	--	35	6,00
Bladder	1 (1,7)	0	1 (1,7)	31,00 --	--	31	8,00
Prostate	1 (1,7)	1 (1,7)	0	47,00 --	--	47	2,00
Unknown	3 (5,0)	2 (3.3)	1 (1,7)	32,67 ± 12,22	2,31–63,02	22–46	5,00 (0–8)
All	60 (100)	26 (43,3)	34 (65,7)	33,50 ± 10,11	30,68–36,03	11–52	5,42 (0–9)

SD: Standard deviation, CI: Confidence interval

A total of 13,3% of all participants had coronavirus infection 3 to 14 months prior to the study (mean = 6,1 months), half of whom did not perceive complete recovery after COVID-19 and reported severe fatigue (VAS ≥ 7) (Fig. 1).

### CRF and function

Most participants (71,6%) had an ECOG level 0 and 1, only one patient had severe dependence or an ECOG level 3 and at the same time was part of the COVID + group; nevertheless, no significant differences in ECOG mean score were observed between COVID + and COVID-19 patients (1,12 vs. 1,05). Patients with status levels 4 and 5 were not included (Table 1).

**Fig. 3:**
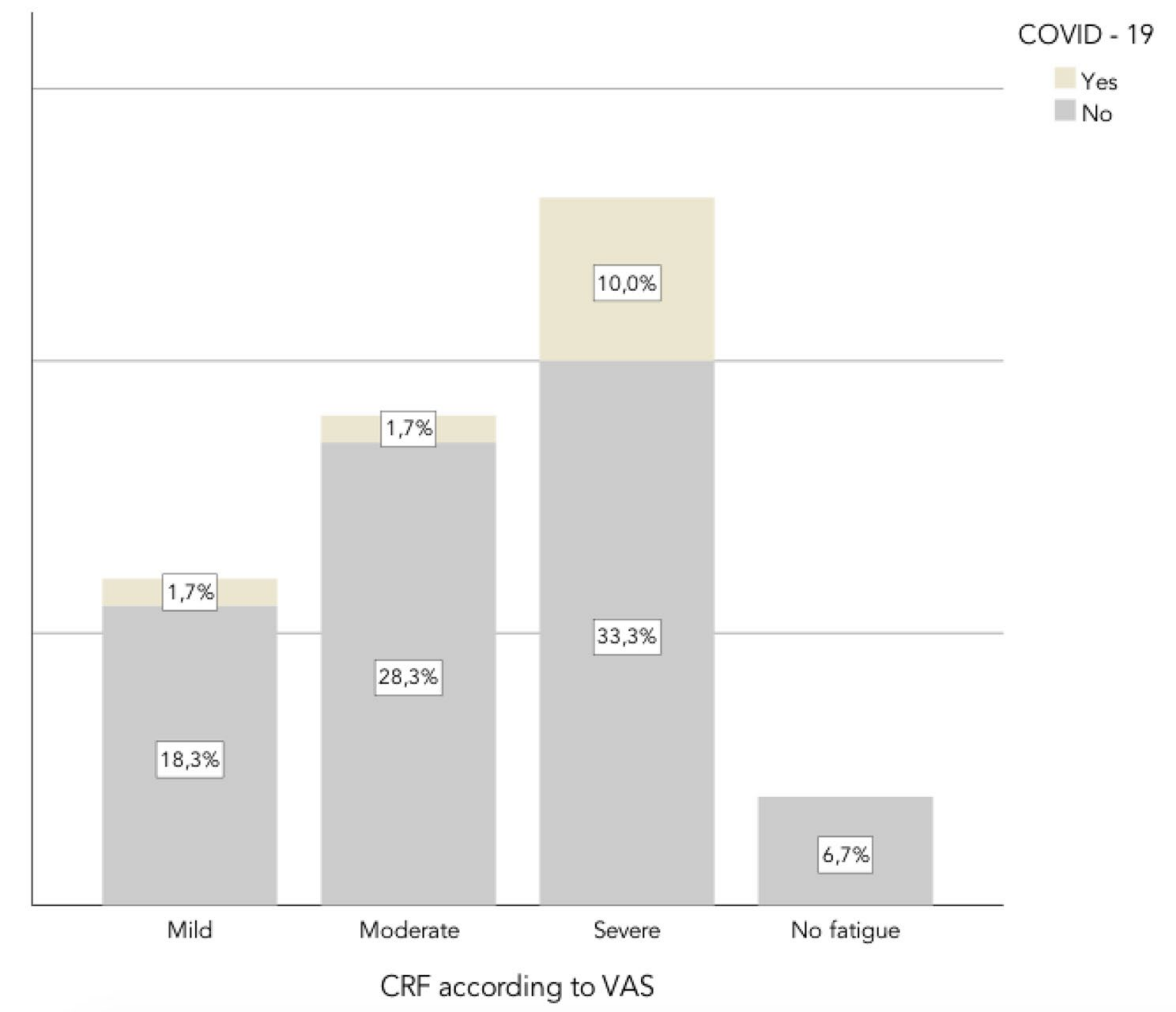
CRF and COVID-19 history

The distribution of performance status according to the primary cancer type is presented in Fig. 2. Regarding the intensity of fatigue measured with the VAS, the mean score was 5,42 [0 a 9]. Only 6.7% had no fatigue symptoms or VAS = 0, 20% had mild fatigue or VAS ≤ 3, 30% had moderate fatigue or VAS 4 to 6, and 43.3% had severe fatigue or EVA ≥ 7 (Fig. 3). The highest VAS scores were found in patients who did not perceive a complete recovery from symptoms after COVID-19 (VAS mean score = 8,25).

The mean FACIT-F score in all cancer patients was 33,5 ± 10,11 [IQR 11–52]. A univariate analysis of factors associated with FACIT-F scores is shown in Table 3. There was a statistically significant difference in the score on the group with previous infection by SARS-CoV-2 (25 ± 10,40) vs. the group without this infection (34,81 ± 9,50); *p* = 0,009. Also, cancer patients with a COVID + history who did not have a perception of recovery after viral infection had an even lower score (FACIT-F = 21,7). Among the results of the FACIT-F scale in certain groups, cancer patients undergoing active immunotherapy treatment (27,5 ± 10,5) and those with a history of depression (24,5 ± 10,5) had a low score, indicating the poorest HRQoL due to CRF, in addition to the COVID + group.

**Table 3:** FACIT- F and VAS outcomes

	*n* (%)	FACIT-F score				VAS of CRF
		Mean ± SD	CI (95%)		Range	Mean(Range)
**All Patients**	60 (100)	33,50 ± 10,11	30,68–36,03		11–52	5,42 (0–9)
**Sex**	*p* = 0,58, t= -0,545 º					
Female	44 (73,3)	33,07 ± 10,23	29,84–36,00		11–52	5,77 (0–9)
Male	16 (26,7)	34,69 ± 10,01	29,57–39,46		19–47	4,44 (0–8)
**SARS CoV-2 infection**	*p* = 0,009, t=-2,684 º					
COVID +	8 (13,3)	25 ± 10,40	18–33,12		11–43	7,12 (3–9)
COVID -	52 (86,7)	34,81 ± 9,50	32,21–37,2		14–52	5,15 (0–9)
**Type of anticancer therapy**	*p* = 0,15, F = 1,80 ∞					
Chemotherapy/Endocrine	23	33,87 ± 9,59	29,72–38,02		14–49	52 (86,7)
Immunotherapy	10	27,10 ± 10,08	19,89–34,31		11–44	6,50 (3–9)
Radiotherapy	4	34,50 ± 11,47	16,25–52, 6		19–45	6,00 (3–8)
Other or unknown	23	35, 74 ± 9,92	31,45–40,03		17–52	4,74 (0–9)
**Comorbidities**	*p* = 0,54, F = 0,72 ∞					
Hypothyroidism	8 (13,3)	32,50 ± 10,78	23,48–41,52		11–45	6,88 (5–9)
Chronic pulmonary disease	3 (5,0)	31,33 ± 10,01	6,45–56,22		21–41	5,33 (3–8)
Depression	2 (3,3)	24,00 ± 5,65	-26,82–74,82		20–28	5,50 (3–8)
**Metastatic disease**	*p* = 0,075, t= -1,811 °					
Yes	26	30,85 ± 11,17	26,65–35,26		11–52	5,73 (0–9)
No	34	35,53 ± 8,86	32,33–38,40		17–51	5,18 (0–9)

SD: Standard deviation, CI: Confidence interval

° T test, ∞ ANOVA

According to the oncologic diagnosis, participants with pancreatic cancer had the highest average of fatigue by VAS = 7,67 and the lowest FACIT_F scores (18,00 ± 3,60);, contrary to colorectal and head and neck cancer, which perceived fewer fatigue symptoms and had the best physical function scores (Table 2).

**Fig. 4:**
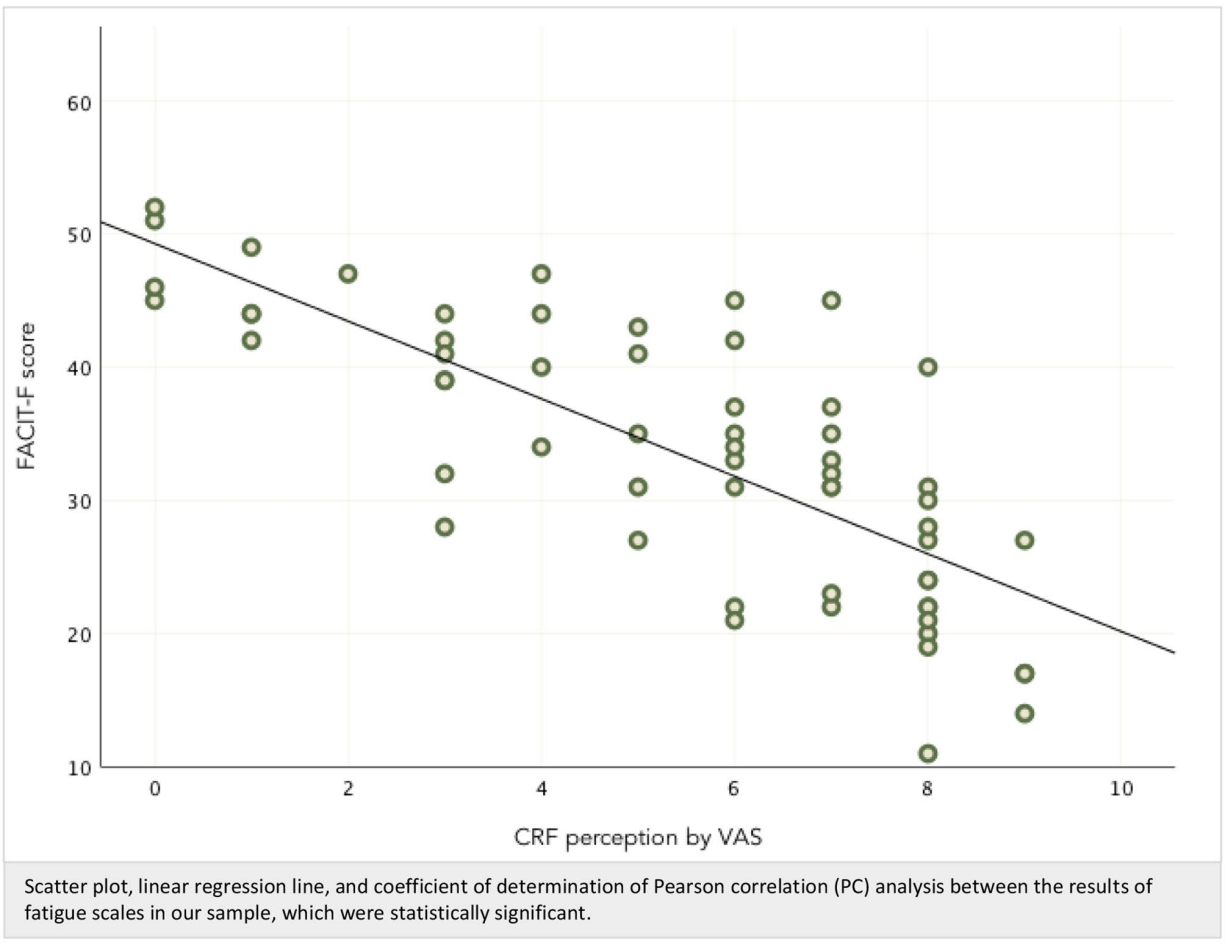
Correlation analysis between FACIT-F and VAS results

## Discussion

Patients with cancer were considered a vulnerable population during the course of the SARS-CoV-2 pandemic. As a principal finding in this population, our study shows a significant difference in the FACIT-F score between the COVID + group and COVID– group (25 ± 10,40 vs. 34,81 ± 9,50 [*p* = 0,009], respectively), which means that cancer patients with a history of coronavirus infection have a major impairment on physical function during their usual daily activities due to CRF compared to cancer patients without coronavirus infection.

As mentioned, in Spain, there is no precise data on the prevalence of COVID-19 infection in the oncology population. However, during the time this study was conducted, 6,128,902 cases were reported in the general population, which corresponds to approximately 13% of the Spanish population. This percentage of COVID + patients aligns with our findings [30].

Nowadays, there is a lack of research evaluating the direct impact of the COVID-19 pandemic on CRF. There are observational studies to describe fatigue following SARS-CoV-2 infection as one of the principal symptoms of post-COVID-19 syndrome with reported values ranging from 52.3 to 72.8% or more [24, 31]. A systematic review and meta-analysis which includes 211 studies on 13 368 074 individual and provides valuable information about the prevalence and risk factors, when comparing COVID-19 patients with non-COVID-19 individuals, were fatigue is one of the most frequently reported persistent symptoms after infection, and factors frequently associated with a higher prevalence of persistent symptoms as female gender, advanced age, comorbidities, an extended duration of hospital stay [32]. However, it is important to note that the individuals analyzed in these studies are part of the general population. By other hand, in most cases the fatigue it was defined as a neurological symptom [33], unlike CRF which is multidimensional.

Specifically in the oncological population, studies such as one involving 2795 patients with cancer who survived COVID-19 documented a 15% range of sequelae, with fatigue (41.0%) and respiratory symptoms (49.6%) being the most common [34]. According to post-COVID-19 syndrome, in our patient group, at least three months had passed between the SARS-CoV-2 infection and the interview. Although, by definition, the presence of cancer would rule out the diagnosis of this syndrome, the high values of fatigue, leads us to reflect on whether SARS-CoV-2 infection in the oncological population could be a risk factor for exacerbation of CRF or a concomitant diagnostic of post-COVID syndrome. However, it is not possible to make this distinction with our data or with the current literature data in the oncological population.

In our study, 93.3% of all cancer patients reported some level of fatigue and according to the VAS of CRF, the CRF in the COVID + group was severe (mean = 7), while in the COVID – group, it was moderate (mean = 5). The VAS values of fatigue were even higher in COVID + cancer patients who did not perceive a complete recovery after coronavirus; It is important to note that the perception of recovery after COVID-19 infection is subjective for each participant, of a dichotomous nature in our questionnaire. Therefore, attributing an incomplete recovery solely to CRF is not possible; other factors, such as the patient’s mood or the presence of other symptoms, may play a role in this observation. Regarding the perception of recovery after coronavirus infection, a descriptive study conducted through an online survey to assess multiple relevant symptoms approximately 3 months after the onset of SARS-CoV-2 infection in 2113 participants revealed that about 80% reported a moderate or poor health status and persistent symptoms, including fatigue and dyspnea, were most prevalent [35], again, in nononcologic patients.

Otherwise, the HRQoL of cancer patients during the pandemic was significantly lower compared with general population [36, 37], as shown in our results. In addition, a European study in cancer patients undergoing chemotherapy found a deteriorated HRQoL due to fatigue and insomnia symptoms [38]. However, none of these results are directly related to viral infection, which means that most studies have evaluated the impact on quality of life as an indirect consequence of COVID-19.

Certainly, the negative impact of CRF on daily function and HRQoL assessment using the FACIT-F scale has been supported in other studies [39–41]. Our participants, according to FACIT-F scores, had an average of 33,5 ± 10,11 (a score ≤ 30 means severe CRF and worst HRQoL); moreover, there were some groups with a major impairment of HRQoL, such as cancer patients with metastatic disease (30,85 ± 11,17), and participants in treatment with immunotherapy had a score of 27,10 ± 10,08, the last group is in keeping with previous studies where CRF has been associated with toxicity and side effects of immunotherapy [42, 43]. Also, cancer patients with a history of depression in our findings had a 24 ± 5,65 FACIT-F score, other research about the impact of the pandemic on cancer people shows deteriorated emotional wellbeing [44, 45] and a significant prevalence of anxiety and loss of energy [46]; additionally a published study with 187 cancer patients reported a high rate of symptoms due to the lockdown, where 55.9% had fatigue at the end of the day, 91.5% cognitive alterations and 78% insomnia [47], which may explain that cognitive or emotional dimensions of CRF were the most affected.

Among the different tumor entities in our study, pancreatic cancer patients had a significantly higher perception of CRF and reported a worse HRQoL (FACIT-F 18,00 ± 3,60; EVA 7,67 [6–9]). Similar results have been obtained in a study examining the prevalence and severity of fatigue in 2244 cancer patients across 15 entities, where CRF levels were significantly higher in pancreatic cancer patient, particularly in the physical dimension [48]; Importantly, it is crucial to note that the entire group of our pancreatic cancer patients presented with metastatic disease, indicating an advanced stage of illness that could significantly influence the levels of experienced fatigue. Apart from the difference groups according to SARS-CoV-2 exposure, the differences in the FACIT-F results above the other variables were not statistically significant and should be interpreted with caution due to low size in some groups.

All the values of the FACIT-F scale obtained in this study had an inverse correlation compared to the VAS values (Pearson’s *r* = – 0,76), which means that the HRQoL level is better meanwhile the perception of asthenia is lower; this concordance between the two scales was also described in other studies [49]; therefore, we think that each of the tests can be applied to reliably assess this symptom in clinical practice.

In terms of performance status, most of the participants had a mild or no dependence for functional ability (ECOG-PS ≤ 2), which could be a protective factor. According to a cohort study on patients with active or previous malignancy and confirmed SARS-CoV-2 infection, an ECOG-PS of 2 or higher was one of the independent risk factors associated with increased 30-day all-cause mortality and morbidity (2 vs. 0 or 1: 3,89, 2,11 − 7,18) [50]. Despite the good performance status in our patients, only 28.3% had an active job, which suggests that the function for basic self-care activities is not as limited as the function for advanced activities, but the information is insufficient to statistically define whether this is related to CRF or the pandemic socioeconomic situation.

On the other hand, there is strong evidence of the beneficial effects of physical activity in CRF [51, 52] and the improvement of cancer health-related outcomes [53], as well recent studies have been remark the importance of rehabilitation programs because home confinement can put cancer patients at a greater risk of physical deconditioning and immobilization [54]; related to this, we think that it is important to analyze that 53.3% of our patients reported regular physical activity, and this value is high compared to other works [55], but it is not possible to know if these percentages can be associated with CRF levels according to the results. While it is true that some effective interventions to enhance adherence to healty lifestyle habits, including exercise, are increasingly employed in people with cancer [56], it is possible that patients in this sample, who also mostly had a good performance status (ECOG levels 0 and 1), took part in these interventions. This, in turn, may explain their interest in actively participating in medical research studies such as this one.

### Strengths and limitations

The current study represents, to the best of our knowledge, the first report in the literature to assess CRF values based on exposure to SARS-CoV-2 infection using validated tools to measure outcomes in terms of HRQoL and performance status (measured by FACIT-F) within the oncology population.

In spite of the methodological efforts to control heterogeneity in the final sample through selection criteria, it is not necessarily considered representative of the overall oncological population; nevertheless, our group of cancer patients is related to the descriptions of the rest of the oncological population in Spain.

Although our work provides new information on the significant impact of COVID-19 and CRF on oncology patients, there are limitations to warrant.

The online application of the questionnaire may be difficult due to the multidimensional aspects of CRF diagnosis, and patients or caretakers may not know their entire clinical history. Also, the participation of people who do not have access to electronic devices and the internet is limited.

Despite the significant impact on social media and the imperative to achieve the calculated sample size, the major limitation arose from the small number of participants, particularly noticeable in certain groups such as the COVID + group. This limitation affected the application of statistical tests for comparing and interpreting some results. Concerning the composition of the patient group, in addition to the presence of metastatic disease, information about the cancer stage was not included in the study, which could also correlate with the levels of fatigue. Similarly, apart from determining the type of oncological treatment the patients were undergoing during data collection, additional details, such immunotherapy medications used, which may influence the clinical course of fatigue were not investigated. Moreover, in our patient sample, to ascertain the diagnostic of depression as a comorbidity that significantly contributes to CRF, it relied on the question of whether there was a history of depression; we recognize that this approach may present a limitation, as values could potentially be higher with the utilization of questionnaires aimed at identifying the actual presence of a depressive disorder.

On the other hand, given that there is a considerable number of asymptomatic SARS-CoV-2 infections, it is possible that some of these patients have been analyzed within the COVID-19 group.

CRF is a neglected and often undiagnosed symptom that warrants further research to highlight its significance, particularly given its high prevalence and substantial impact on both cancer patients and their families. In this regard, we consider the observational results of this study to offer valuable and original insights, shedding light on the heightened fatigue symptoms and diminished physical function experienced during routine daily activities post-COVID-19. Finally, owing to its rigorous methodology, this study is reproducible, its limitations are identifiable for potential enhancement, and it contributes to the augmentation of high-quality evidence in related research endeavors.

## Conclusion

According to the results of our research, we identified that exposure to SARS-CoV-2 infection increases symptoms of CRF and impacts physical function for ADLs, thus worsening. Notwithstanding the small size of the COVID + group and the knowledge that fatigue perception is already high among cancer patients, our study emphasizes the importance of recognizing specific most vulnerable groups or variables within this population that further elevate CRF, conditioning a lower HRQoL. The high correlation between the HRQoL by FACIT-F results and the patient perception of fatigue in our work is also notable, which positively values the properties of this scale and highlights the value of employing validated tools for diagnosis and follow-up in clinical practice.

CRF (Cancer-Related Fatigue) warrants further research to underscore its significance, and these early observations highlight an emerging issue in cancer patients during the pandemic; we expect that health-care personnel will be informed about diagnostic strategies for this symptom, allowing for timely intervention, especially when there is suspicion of worsening fatigue symptoms after SARS-CoV-2 infection.

As the pandemic evolves, longer-term follow-up and larger sample sizes are needed to understand the effect of SARS-CoV-2 more completely on outcomes in CRF, followed by research on potential treatment options, all aimed at improving care for these vulnerable groups and their families.

### Electronic supplementary material

Below is the link to the electronic supplementary material.

Supplementary Material 1

## Data Availability

The datasets generated and/or analysed during the current study are available from the corresponding author on reasonable request. A sample version of the questionnaire developed for this study is avaliable.
