# Supplementary material for: Cancer-related fatigue and activities of daily living: lessons learned from the COVID-19 pandemic
Source: BMC Palliat Care. 2024 Apr 27;23:110. doi: 10.1186/s12904-024-01437-z (PMC11055275; doi:10.1186/s12904-024-01437-z)
Supplement: Supplementary file 1 — Supplementary Material 1 [file 12904_2024_1437_MOESM1_ESM.docx]

| **Questionnaire on symptoms of fatigue during the Covid-19 pandemic** | | | | | | |
| --- | --- | --- | --- | --- | --- | --- |
| **Information Sheet and Consent** | | | | | | |
| Fatigue that occurs in people with cancer: "cancer-related fatigue " is define as the experience of physical, emotional, and/or mental exhaustion related to cancer or its treatment. Fatigue is not the typical tiredness that follows an active or long day; it is a lack of energy that is distressing, does not improve with normal amounts of rest or sleep and disrupts activities of daily life. On the other hand, it has also been observed that some people who have suffered from COVID-19 have persistent fatigue as a consequence.  The current pandemic has changed the way we live, mainly in some groups at high risk of complications such as cancer patients; where a more detailed understanding of the impact of COVID-19 is needed in order to provide the best treatments and timely measures to preserve quality of life.    This study in Spanish oncology population, was designed to assess cancer-related fatigue symptoms and their implications on physical function and quality of life during after coronavirus infection using this questionnaire. It has the approval of the Research Ethics Committee of the Universidad Pontificia Comillas and the Hospital Clínico Universitario San Carlos de Madrid.  Whatever your situation, you can complete this questionnaire, it is not a requirement to have been COVID positive to fill it out. It consists of 18 questions, with a scheduled completion time of approximately 10 minutes. Your participation is voluntary, in addition, all the information you provide us is confidential, this means that it will only be used for the purposes of this research and will be operated by the researchers. Your answers will not influence your current treatment. Your personal data will not be requested.    Your contribution is very important, and we appreciate your collaboration. If you have any questions, contact with the corresponding author Iveth Urbano: 202006265@alu.comillas.edu | | | | | | |
| **I have read the description and give my consent to participate:**   - **Yes** | | | | | | |
| **Section 1. Questions about you:** | | | | | | |
| **1. Sex:**   - Female - Male - Prefer not to say | | | | | | |
| **2. What is your birth date?**        Day/Month/Year | | | | | | |
| **3. What is your current employment status?**   - Currently working - Not employed - Retired - Studying | | | | | | |
| **4. Place of medical care:**   - Urban - Rural | | | | | | |
| **5. Living arrangements:**   - Lives with family - Lives alone - Lives in a nursing home or hospice | | | | | | |
| **6. Have you been diagnosed with any of these diseases? Select all that apply.**   - Anemia - Dementia - Depression - Cardiac disease - Pulmonary disease - Hypothyroidism - None | | | | | | |
| **7. How much physical activity have you done in the last year?**   - More than 150 minutes per week - Less than 150 minutes per week - None | | | | | | |
| **8. Are you currently being treated, or have you been treated in the last month with any of these medications? Select all that apply.**   - Methylphenidate - Methylprednisolone - Megestrol acetate - Erythropoietin - Dexamethasone - Modafinil - L-Carnitine - None | | | | | | |
| **9. Do you know where your PRIMARY DISEASE is located?** Primary disease is the organ where the tumor started to grow: | | | | | | |
| - Head and neck - Breast - Lung - Brain - Esophagus - Stomach - Colorectal - Pancreas - Liver/Bile duct - Prostate | | - Ovary - Cervix - Uterus - Kidney - Bladder - Genitals - Skin/Melanoma - Leukemia / Multiple myeloma / Lymphoma - Bones/Osteosarcoma - Sarcoma - Not know | | | | |
| **10. Have you been diagnosed with metastatic disease?** Metastasis is the presence of malignant tumours in other regions beside the primary location.   - Yes - No | | | | | | |
| **11. What is your current type of anticancer therapy? Select all that apply to this last year:**   - Chemotherapy - Immunotherapy - Endocrine - Radiotherapy - Surgery - None/ Not know | | | | | | |
| **12. Have you been diagnosed with COVID-19?**   - Yes - No | | | | | | |
| **13. If the previous question was: YES. Do you consider yourself recovered from your symptoms after COVID-19?**   - Yes - No | | | | | | |
| **14 When was the date of COVID-19 diagnosis?**        Day/Month/Year | | | | | | |
| **Section 2** | | | | | | |
| **15. PERCEPTION OF FATIGUE**  **In the last month, have you experienced fatigue? (Physical, emotional and/or mental exhaustion during the activities of daily living). Please rate the intensity of your symptom below, on scale of 0 to 10.**  🞆 0        🞆 1        🞆 2        🞆 3        🞆 4        🞆 5        🞆 6       🞆7        🞆 8        🞆9        🞆10 | | | | | | |
| **16. ECOG performance**  **Based on your performance in activities of daily living, select the group that best describes your own level:**   - Fully active, able to carry on all pre-disease performance without restriction. - Restricted in physically strenuous activity but ambulatory and able to carry out work of a light or sedentary nature, e.g., light house work, office work. - Ambulatory and capable of all selfcare but unable to carry out any work activities; up and about more than 50% of waking hours. - Capable of only limited selfcare; confined to bed or chair more than 50% of waking hours. - Completely disabled; cannot carry on any selfcare; totally confined to bed or chair | | | | | | |
| **17. FACIT Fatigue Scale**  Below is a list of statements that other people with your illness have said are important. Please circle or mark one number per line to indicate your response as it applies to the past 7 days. | | | | | | |
|  | **Not at all** | | **A little bit** | **Somewhat** | **Quite a bit** | **Very much** |
| I feel fatigued | 🞆 | | 🞆 | 🞆 | 🞆 | 🞆 |
| I feel weak all over | 🞆 | | 🞆 | 🞆 | 🞆 | 🞆 |
| I feel listless (“washed out”) | 🞆 | | 🞆 | 🞆 | 🞆 | 🞆 |
| I feel tired | 🞆 | | 🞆 | 🞆 | 🞆 | 🞆 |
| I have trouble starting things because I am tired | 🞆 | | 🞆 | 🞆 | 🞆 | 🞆 |
| I have trouble finishing things because I am tired | 🞆 | | 🞆 | 🞆 | 🞆 | 🞆 |
| I have energy | 🞆 | | 🞆 | 🞆 | 🞆 | 🞆 |
| I am able to do my usual activities | 🞆 | | 🞆 | 🞆 | 🞆 | 🞆 |
| I need to sleep during the day | 🞆 | | 🞆 | 🞆 | 🞆 | 🞆 |
| I am too tired to eat | 🞆 | | 🞆 | 🞆 | 🞆 | 🞆 |
| I need to help doing my usual activities | 🞆 | | 🞆 | 🞆 | 🞆 | 🞆 |
| I am frustrated by being too tired to do the things I want to do | 🞆 | | 🞆 | 🞆 | 🞆 | 🞆 |
| I have to limit my social activity because I am tired | 🞆 | | 🞆 | 🞆 | 🞆 | 🞆 |

Link to a sample version of the digital questionnaire:

<https://forms.office.com/Pages/ResponsePage.aspx?id=HHDSvJuqEk26IPPjuDBwwbAQwXs85vZCj3aJ4jVrOyJUOTlLWklISTQxREI5QkxJVFI2OFczVDBBMC4u>
